# Graphene Oxide and Reduced Graphene Oxide Exhibit Cardiotoxicity Through the Regulation of Lipid Peroxidation, Oxidative Stress, and Mitochondrial Dysfunction

**DOI:** 10.3389/fcell.2021.616888

**Published:** 2021-03-18

**Authors:** Jian Zhang, Hong-Yan Cao, Ji-Qun Wang, Guo-Dong Wu, Lin Wang

**Affiliations:** Department of Cardiovascular Center, The First Hospital of Jilin University, Changchun, China

**Keywords:** graphene family nanomaterials, cardiotoxicity, lipid peroxidation, oxidative stress, mitochondrial dysfunction

## Abstract

**Objective:**

Graphene has been widely used for various biological and biomedical applications due to its unique physiochemical properties. This study aimed to evaluate the cardiotoxicity of graphene oxide (GO) and reduced GO (rGO) *in vitro* and *in vivo*, as well as to investigate the underlying toxicity mechanisms.

**Methods:**

GO was reduced by gamma irradiation to prepare rGO and then characterized by UV/visible light absorption spectroscopy. Rat myocardial cells (H9C2) were exposed to GO or rGO with different absorbed radiation doses. The *in vitro* cytotoxicity was evaluated by MTT assay, cell apoptosis assay, and lactate dehydrogenase (LDH) activity assay. The effects of GO and rGO on oxidative damage and mitochondrial membrane potential were also explored in H9C2 cells. For *in vivo* experiments, mice were injected with GO or rGO. The histopathological changes of heart tissues, as well as myocardial enzyme activity and lipid peroxidation indicators in heart tissues were further investigated.

**Results:**

rGO was developed from GO following different doses of gamma irradiation. *In vitro* experiments in H9C2 cells showed that compared with control cells, both GO and rGO treatment inhibited cell viability, promoted cell apoptosis, and elevated the LDH release. With the increasing radiation absorbed dose, the cytotoxicity of rGO gradually increased. Notably, GO or rGO treatment increased the content of ROS and reduced the mitochondrial membrane potential in H9C2 cells. *In vivo* experiments also revealed that GO or rGO treatment damaged the myocardial tissues and changed the activities of several myocardial enzymes and the lipid peroxidation indicators in the myocardial tissues.

**Conclusion:**

GO exhibited a lower cardiotoxicity than rGO due to the structure difference, and the cardiotoxicity of GO and rGO might be mediated by lipid peroxidation, oxidative stress, and mitochondrial dysfunction.

## Introduction

In recent years, nanomaterials have been reported to be promising functional materials and display great application potentials in various fields, such as materials, communication, energy, and biomedicine (Guozhong, 2004). Notably, carbon nanomaterials have attracted tremendous attentions due to their unique structural and mechanical properties (Hong et al., 2015).

As a “wonder material,” graphene is composed of single-layer sheet-like and two-dimensional carbon atoms with sp2 hybridized hexagonal honeycomb structure (Choi and Lee, 2016). Currently, it has been applied in biomedical fields, including drug delivery (Yang et al., 2015), cellular imaging (You et al., 2015), solid/liquid phase microextraction (Rezaeifar et al., 2016), and cancer therapy (Rahman et al., 2015; Krasteva et al., 2019). Recently, a variety of graphene-derived nanomaterials (GFNs), such as graphene oxide (GO) and reduced GO (rGO), have attracted a lot of interest in biomedical applications due to their exceptional physical and chemical properties, including good thermal stability, excellent mechanical strength, and high electronic conductivity (Zhang et al., 2016; Papageorgiou et al., 2017). GO, as an oxygenated derivative of graphene, contains a series of active oxygen-containing groups, and is usually prepared by treating graphene with a strong acid or a strong oxidant (Joshi et al., 2015). Different from GO, rGO is the reduced product of GO containing less oxygen-containing groups that developed by several reducing agents (Alam et al., 2017). The difference of GO and rGO in the content of oxygen-containing groups results in different surface properties, such as surface charge, electrical conductivity, and hydrophobicity (Chatterjee et al., 2014).

Unfortunately, despite the biological effects of graphene, many studies showed the cytotoxic effects of nanomaterials (Rahman et al., 2015; Gurunathan and Kim, 2016). The interaction of graphene-based materials with the cell membrane is one of the main causes of cytotoxicity (Seabra et al., 2014; Gurunathan and Kim, 2016). Graphene materials could interact with membrane lipids and receptors, interfere cell metabolism, induce stress, or cause cell death (Jarosz et al., 2016). Moreover, graphene materials could cause oxidative stress and reactive oxygen species production and affect cell DNA integrity and mitochondrial activity (Jarosz et al., 2016). The toxic effect of graphene highly depends on the experimental conditions, such as time of treatment, dose and type of the cells (Zhang et al., 2012; Gurunathan et al., 2013; Seabra et al., 2014).

At present, there is very limited knowledge regarding the cardiotoxicity of graphene and its derivatives. A previous *in vitro* study using rat H9c2 cells indicated that graphene oxide nanomaterials caused mitochondrial disturbances, generation of reactive oxygen species, and DNA damages, but the *in vivo* effect is lacking (Arbo et al., 2019). In addition, an *in vivo* study in zebrafish provided valuable information about the cardiotoxic effects of GO in embryonic development (Bangeppagari et al., 2019). This study found that low concentrations of GO did not show side effects on health. However, GO at high concentrations induced significant embryonic mortality, increase heartbeat, delayed hatching, cardiotoxicity and cardiovascular defects, and increased apoptosis (Bangeppagari et al., 2019). Therefore, it is essential to focus on biosafety and reveal the mechanisms of graphene cytotoxicity.

To date, the toxicity of GO and rGO on cardiomyocytes and tissues has not been well-investigated. In the present study, we prepared rGO by gamma irradiation of GO and evaluated the cardiotoxicity and related mechanisms of GO and rGO *in vitro* and *in vivo*. This study provided deeper understanding of the environmental risks associated with GO and its derivatives on human health in general.

## Materials and Methods

### Characterization of GO

GO dispersion liquid was purchased from XFNANO Materials Tech Co. Ltd. (Nanjing, Jiangsu, China). The shape and thickness of GO were evaluated by atomic force microscopy (AFM, JPK Instruments AG, Berlin, Germany).

### Preparation and Characterization of rGO

GO was reduced by gamma irradiation to prepare rGO. GO dispersion liquid was diluted to 1 mg/mL with ultrapure water and transferred into centrifuge tubes. Next, the tube was injected with nitrogen for 10 min to remove air and then immediately sealed. Afterward, the tubes received ^60^Co (45,000 Curie) gamma irradiation with different absorbed doses (0, 50, 100, 200, 300, 400 kGy, namely, GO, r50GO, r100GO, r200GO, r300GO, and r400GO) at the irradiation dose rate of 2 kGy/h at room temperature, and the irradiated GO was sonicated in a water bath for 10 min. Subsequently, 100 μL of irradiated GO was transferred into a transparent centrifuge tube containing 900 μL of ultrapure water, and sonicated for 30 min. The color change of GO after gamma irradiation was observed by photography. In addition, the absorption spectra of GO underwent different doses of gamma irradiation were measured by UV/visible light absorption spectroscopy (Biochrom, Cambridge, United Kingdom).

### Cell Culture and Treatment

Rat myocardial cells (H9C2) were obtained from Shanghai Obio Technology Co., Ltd., and maintained in complete DMEM medium (Gibco, Carlsbad, CA, United States) at 37°C under 5% CO2. H9C2 cells were treated with 50 μg/mL of GO, r50GO, r100GO, r200GO, r300GO, and r400GO, respectively, for 24 h. The cellular morphology was observed under light microscope.

### Cell Viability Assay

H9C2 cells were grown in 96-well plates and then underwent the above treatments for 24 and 48 h. Each well was added with 10 μl of MTT (5 mg/mL, Sigma) for another 4 h. Afterward, 100 μL of dimethyl sulfoxide was added. Microplate spectrophotometer (Thermo Fisher Scientific, Middletown, VA, United States) was used to evaluate cell viability based on the absorbances at 470 nm.

### Lactate Dehydrogenase (LDH) Detection

H9C2 cells were grown in 96-well plates and then underwent the above treatments for 24 and 48 h. Cultural supernatants were collected to detect LDH release by the commercially available kit (Nanjing Jiancheng Bio Inst, China) according to the manufacturer’s instructions. Absorbance at 340 nm was used to calculate LDH release by a microplate spectrophotometer (Thermo Fisher Scientific, Middletown, VA, United States).

### Cell Apoptosis Assay

FITC-Annexin V Apoptosis kit was used to detect apoptosis. H9C2 cells underwent the above treatments for 24 h. Cells were dislodged and harvested using trypsin, rinsed with PBS, and resuspended with Binding Buffer. After incubation with FITC-Annexin V and PI for 15 min, flow cytometry (BD, CA, United States) was used to calculate the number of apoptotic cells.

### Measurement of Reactive Oxygen Species (ROS)

Oxidative damage indicator ROS content in H9C2 cells with various treatments was determined using the total ROS assay kit based on DCFH-DA staining (Beyotime, Beijing, China). Mitochondrial specific ROS was measured using the MitoSox staining kit (Beyotime, Beijing, China). DAPI was used for staining of cell nucleus. The cells were observed under inverted fluorescence microscope (Olympus, Japan).

### Mitochondrial Membrane Potential Detection

Mitochondrial membrane potential depolarization was detected using commercial JC-1 kit (Beyotime). At high membrane potential, JC-1 shows red fluorescent aggregates, while JC-1 presents green fluorescent monomers at low membrane potential. Briefly, cells were collected and stained with 1 ml of JC-1 dye at 37°C for 20 min. Next, cells were rinsed with JC-1 buffer, and observed under an inverted microscope (Olympus, Japan).

### Animal Experiment

Approval from the local animal Ethics Committee of the First Hospital of Jilin University was obtained prior to experiments [Number (201804-003)], and the experiments were carried out following the Guide for the Care and Use of Experimental Animals. A total of 40 healthy C57BL/6 male mice (8 weeks old, purchased from Charles River, Beijing, China) were used for the following experiments after 1 week of acclimation. Forty mice were randomly and equally assigned into four groups: control group (*n* = 10), GO group (*n* = 10), r200GO group (*n* = 10), and r400GO group (*n* = 10). Mice in the GO, r200GO, and r400GO groups were injected with 4 mg/kg GO, r200GO, and r400GO *via* tail vein, respectively. Mice in the control group were injected with saline solution. After 14 days of injection, mice were euthanized by CO_2_ asphyxiation, and the heart tissues were collected and weighed. The heart weight coefficient was calculated as the ratio of heart wet weight (g)/body weight (g)^∗^100. For mitochondrial membrane potential, cardiac tissue was filtered through a cell strainer (BD Biosciences, Bedford, MA) and centrifuged (1,500 rpm, 10 min) to obtain single-cell suspension for flow-cytometry based assays.

### Measurements of Myocardial Enzyme Activity and Lipid Peroxidation Indicators in Heart Tissues

The heart tissues were homogenized, and the activities of myocardial enzymes, including LDH, aspartate aminotransferase (AST), Na^+^/K^+^-ATPase, and Ca^2+^/Mg^2+^-ATPase, were detected using commercial kits (Nanjing Jiancheng). In addition, lipid peroxidation indicators, including malondiadehyde (MDA), superoxide dismutase (SOD), glutathione peroxidase (GSH-Px), and total antioxidant capacity (T-AOC), in heart tissues were determined using commercial kits (Nanjing Jiancheng).

### Immunostaining

Tissue sections of 5-μm thickness were prepared from formalin-fixed paraffin-embedded tissues. After deparaffinization, sections were washed in distilled water and permeabilized in 0.1% Triton X-100. The sections were used for *in situ* detection of apoptosis by TUNEL using TdT *In Situ* Apoptosis Detection Kit (R&D systems). For immunofluorescence staining, following primary antibody (anti-troponin I, abcam, #ab47003) overnight at 4°C, sides were washed and incubated with the secondary fluorescence-labeled antibody for 1 h in the dark at room temperature and then counterstained using DAPI. Slides were observed under an inverted microscope (Olympus, Japan).

### Statistical Analysis

Data were presented as the mean ± SD. One-way ANOVA followed by multiple comparisons was used for data comparisons using SPSS software. *P* < 0.05 was considered significant.

## Results

### Characterization of GO and rGO

**FIGURE 1:**
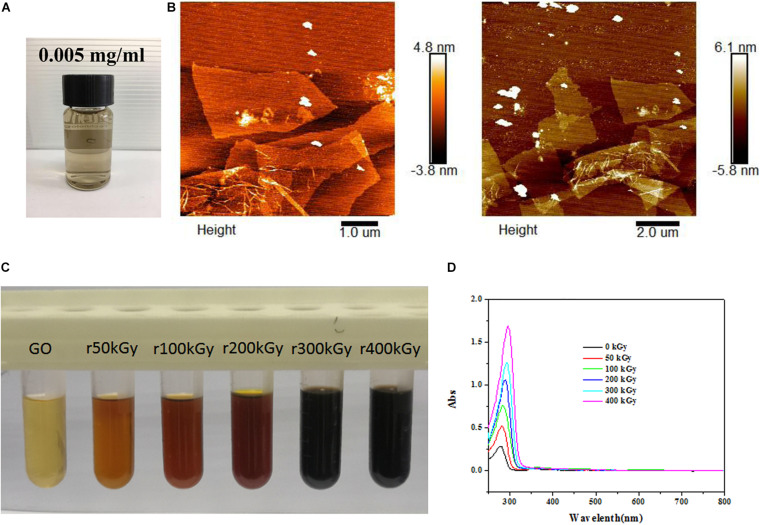
Characterization of graphene oxide (GO) and reduced GO (rGO). **(A)** The appearance of commercial GO dispersion liquid. **(B)** The representative images of GO using atomic force microscopy. **(C)** The colors of GO exposed to different radiation absorbed doses. **(D)** The ultraviolet visible absorption spectra of GO exposed to different radiation absorbed dose.

Commercial GO dispersion liquid exhibited yellow-brown appearance (Figure 1A). As determined by AFM, GO showed 3–4 nm of thickness and 100–200 nm of diameter (Figure 1B), suggesting the formation of single or few layers of GO. After gamma irradiation, the color of GO changed from pale yellow to brown, and finally to black with the increasing radiation absorbed dose (Figure 1C). Meanwhile, optical absorption spectra revealed that the wavelength of GO was also increased with the increasing radiation absorbed dose (Figure 1D), which suggested that GO was reduced by gamma irradiation as rGO.

### Effect of GO and rGO on Cytotoxicity in H9C2 Cells

**FIGURE 2:**
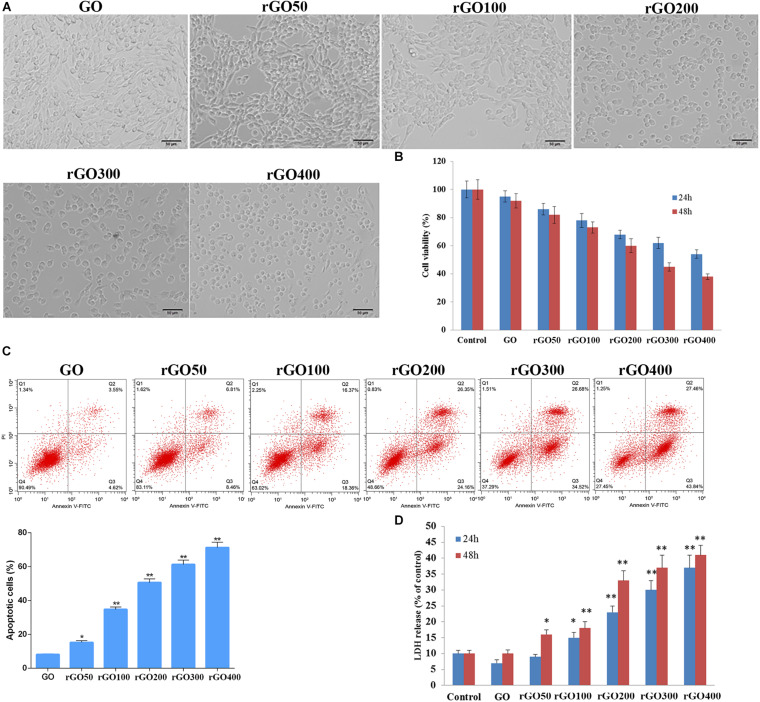
Cytotoxicity of graphene oxide (GO) and reduced GO (rGO) in H9C2 cells. **(A)** Cell morphology when H9C2 cells treated with GO or rGO. **(B)** Cell viability of H9C2 cells treated with GO or rGO at 24 and 48 h using MTT assay (*n* = 3). **(C)** Cell apoptosis rate of H9C2 cells treated with GO or rGO at 24 h using flow cytometry analysis (*n* = 3). **(D)** Lactate dehydrogenase (LDH) release of H9C2 cells treated with GO or rGO at 24 and 48 h using the commercial kit (*n* = 3). *p < 0.05; **p < 0.01.

As shown in Figure 2A, GO-treated H9C2 cells exhibited the normal morphology and reached near 100% confluence, while rGO-treated H9C2 cells exhibited abnormal morphology and lower cell numbers with the increasing radiation absorbed dose. MTT assay showed that compared with control cells, GO or rGO obtained with increasing radiation-absorbed doses inhibited cell viability at 24 and 48 h (Figure 2B). Consistently, flow cytometry analysis found that the rate of apoptotic cells was gradually increased after GO or rGO treatment with the increasing radiation absorbed dose in H9C2 cells (Figure 2C). In addition, LDH release was detected to further determine cytotoxicity, and the results showed that compared with control cells, GO has little effects on H9C2 cells, while rGO generated with the increasing radiation absorbed dose gradually enhanced the LDH release (*p* < 0.05, Figure 2D).

### Effect of GO and rGO on Oxidative Damage and Mitochondria Damage in H9C2 Cells

**FIGURE 3:**
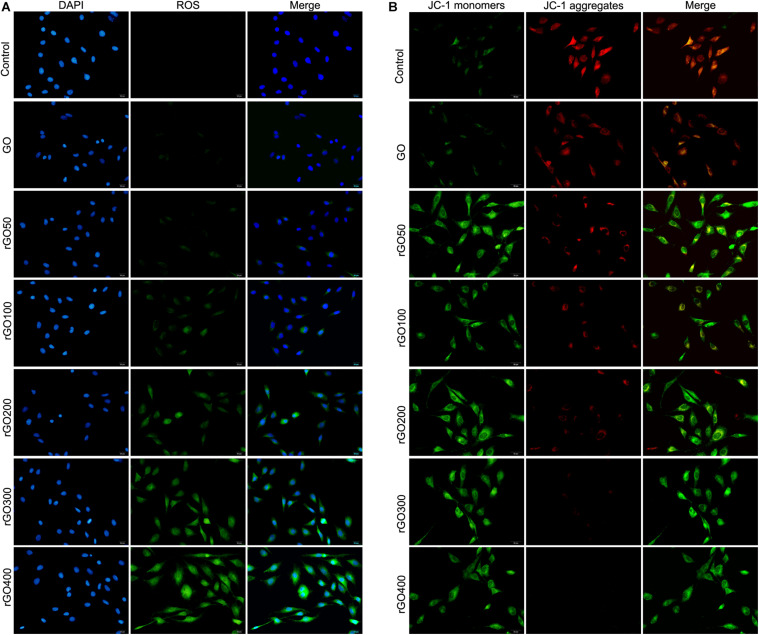
Graphene oxide (GO) and reduced GO (rGO) induced oxidative damage and mitochondria damage in H9C2 cells. **(A)** Reactive oxygen species (ROS) content in H9C2 cells treated with GO or rGO using the DCFH-DA staining kit. **(B)** The mitochondrial membrane potential in H9C2 cells treated with GO or rGO.

**FIGURE 4:**
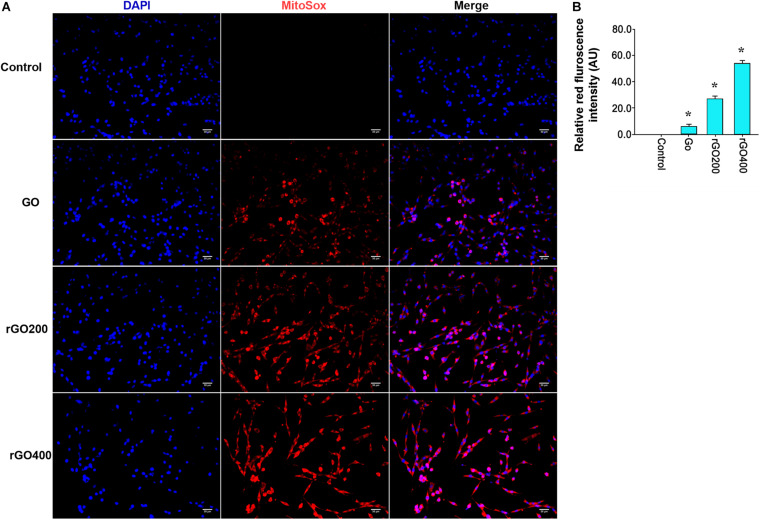
ROS was mainly generated through mitochondria. **(A)** Representative MitoSox staining of GO and rGO treated H9C2 cells. DAPI was used to stain nucleus. **(B)** The relative MitoSox intensity of the control and treated cells (*n* = 3). *p < 0.05.

Oxidative damage indicator ROS was measured to evaluate cellular oxidative damage after GO or rGO treatment in H9C2 cells. We found that H9C2 cells with GO or rGO treatment exhibited gradually increasing total ROS content with the increasing radiation-absorbed dose, as detected by DCFH-DA staining (Figure 3A). In addition, the mitochondrial membrane potential was used to evaluate mitochondria damage, and the results showed that control cells mainly presented JC-1 aggregates (red fluorescence), while increased JC-1 monomers (green fluorescence) and reduced JC-1 aggregates were observed in cells treated with GO or rGO treatment with the increasing radiation absorbed dose (Figure 3B). We analyzed the source of ROS using a mitochondria-specific MitoSox staining kit and found that ROS was mainly generated in mitochondria after GO or rGO treatment (Figure 4A). rGO400 induced the highest ROS production compared to GO and rGO200 (Figure 4B).

### Effect of GO and rGO on Myocardial Tissues in Mice

**FIGURE 5:**
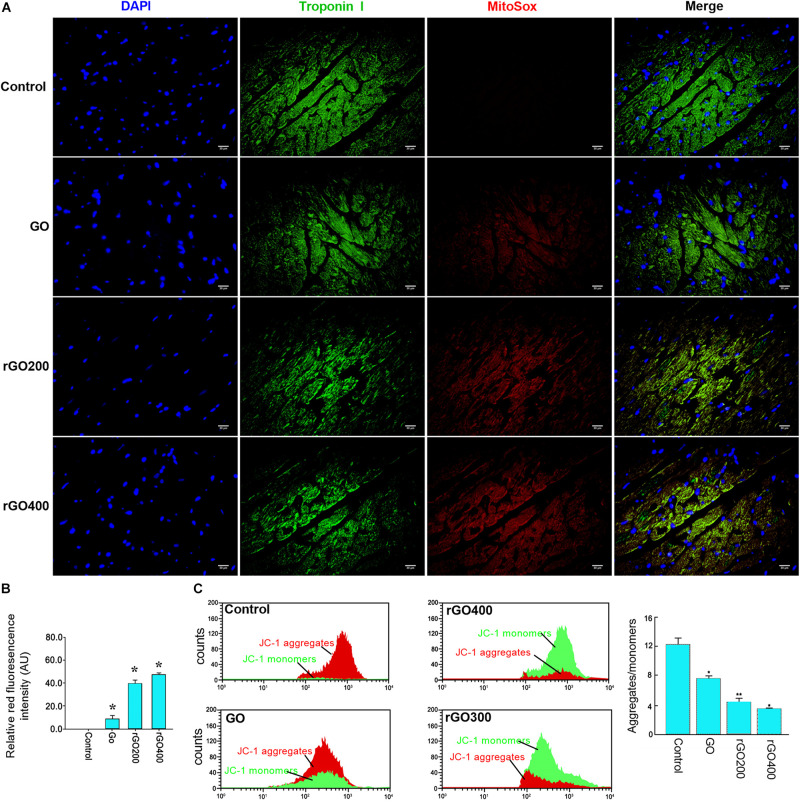
*In vivo* mitochondrial ROS generation and membrane potential in mice heart section. **(A)** The representative MitoSox staining of the heart sections from the control and GO- and rGO-treated mice. Troponin I was used as a cardiac marker. **(B)** The relative MitoSox staining intensity of mouse heart section (*n* = 2). **(C)** Representative flow cytometry analysis of the ratio of JC-1 monomers and JC-1 aggregates in mouse heart tissues (left) and the quantified bar graph (right) (*n* = 3). *p < 0.05; **p < 0.01.

**FIGURE 6:**
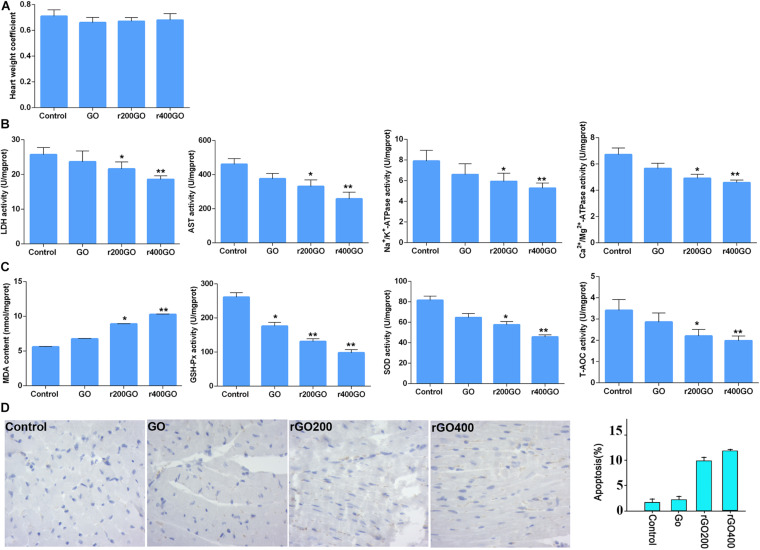
Graphene oxide (GO) and reduced GO (rGO) exerted cardiotoxicity in mice. **(A)** The heart weight coefficient of mice after GO or rGO injection (*n* = 10 in each group). **(B)** The activities of several myocardial enzymes, including lactate dehydrogenase (LDH), aspartate aminotransferase (AST), Na^+^/K^+^-ATPase, and Ca2^+^/Mg2^+^-ATPase, in the myocardial tissues after GO or rGO injection (*n* = 10 in each group). **(C)** The activities of lipid peroxidation indicators, including malondiadehyde (MDA), superoxide dismutase (SOD), glutathione peroxidase (GSH-Px), and total antioxidant capacity (T-AOC), in the myocardial tissues after GO or rGO injection (*n* = 10 in each group). **(D)** The apoptosis of cardiac cells in mice heart section as detected by TUNEL assay. Representative images were shown on the left, and the quantified bar graph was shown on the right (*n* = 3). *p < 0.05; **p < 0.01.

To test the *in vivo* effects, mice were administrated with GO and rGO through tail vein injection. Mitochondrial ROS production and membrane potentials were analyzed after a 14-day treatment. We found that GO and rGO induced significant ROS production compared with control as shown by MitoSox staining of heart tissues (Figures 5A,B), and the mitochondrial function was damaged as shown by the membrane potential changes (Figure 5C). The effects were strong in the rGO-treated group than the GO group. We also analyzed the myocardial functions of the treated mice and found that there was no significant difference in heart weight coefficient after GO or rGO treatment (Figure 6A). In addition, the activities of several myocardial enzymes, including LDH, AST, Na^+^/K^+^-ATPase, and Ca2^+^/Mg2^+^-ATPase, were gradually reduced in the myocardial tissues after GO or rGO treatment with the increasing radiation absorbed dose (Figure 6B). The lipid peroxidation indicator MDA content was gradually increased, while the activities of GSH-Px, SOD, and T-AOC showed a decreasing trend after GO or rGO treatment in myocardial tissues with the increasing radiation absorbed dose (*p* < 0.05, Figure 6C). To see if GO or rGO increase the apoptosis, TUNEL assay of the heart section was performed and we found increased TUNEL positivity in myocardial cells of GO and rGO treated mice, especially in the rGO400-treated group (Figure 6D). Overall, these data suggest GO and rGO exhibited similar cardiotoxicity in mice, similarly as in H9C2 cells.

## Discussion

An exceptional array of physicochemical properties has attracted the attention of many researchers to explore the biomedical applications of graphene, while the occupational or environmental exposure arouses concerns about the following potential risks. To date, the toxicity of GO and rGO on cardiomyocytes and tissues has not been well-investigated. In the current study, rGO was developed from GO following different doses of gamma irradiation. *In vitro* experiments showed that compared with control cells, both GO or rGO treatment gradually inhibited cell viability, promoted cell apoptosis, and elevated the LDH release with the increasing radiation absorbed dose in H9C2 cells. Notably, GO or rGO treatment increased the content of ROS and lowered the mitochondrial membrane potential in H9C2 cells. Moreover, *in vivo* experiments also revealed that GO or rGO treatment damaged the myocardial tissues and changed the activities of several myocardial enzymes and lipid peroxidation indicators in the myocardial tissues.

Both GO and rGO are common derivatives of graphene that possess exceptional physical and chemical properties (Li and Kaner, 2008). As a highly oxidized form of graphene, GO is usually developed by oxidation of graphene using various oxidant agents based on chemical methods, characterized by carboxyl functional groups on plane edges as well as hydroxyl and epoxide functional groups on the basal surface (Papageorgiou et al., 2017). Due to the increased OH and COOH functional groups, GO is easily cross-linked to a variety of materials, including quantum dots, DNA, protein, biomolecules, or polymers, which prevent aggregation in salt and other biological solutions and improve biocompatibility (Kim et al., 2013). Notably, researchers also have focused on the reduction of GO to rGO by various reducing agents, such as sodium borohydride, hydroquinone, methanol, and dimethylhydrazine. However, high toxicity of these reducing agents limits their applications (Pei and Cheng, 2012). Interestingly, ionizing radiation technique, which is environmental friendly and easily operated, has also been proved to be able to induce the reduction of GO (Tuyen et al., 2016; Nguyen et al., 2017; Enayati et al., 2019). In this study, a different dose of gamma irradiation was used to induce the reduction of GO, and rGO was successfully generated according to the results of UV visible light absorption spectroscopy.

Considerable evidence has demonstrated that GO and rGO can be widely used for various biological and biomedical applications due to its unique physiochemical properties (Syama and Mohanan, 2016). Previous studies have demonstrated the antitumor effect and antibacterial activity of GO and rGO (Szunerits and Boukherroub, 2016; Kavinkumar et al., 2017; Zuchowska et al., 2017). However, the biocompatibility and safety of GO and rGO on the human health are still considered as key factors in biomedical applications (Seabra et al., 2014). Cytotoxicity studies of GO and rGO have been conducted in several *in vitro* experiments. Gurunathan et al. (2013) have reported that GO can inhibit cell viability in a dose-dependent manner in MCF-7 cells. GO with a dose >60 μg/mL exerts significant cytotoxicity. Zhou et al. (2014) have evaluated the toxicity of GO in different cancer cell lines, such as mouse melanoma B16F10 cells, prostate cancer PC3 cells, and breast cancer MDA-MB-231 cells, and the results exhibit the cytotoxicity of GO to these cancer cells in a dose-dependent manner. Jaworski et al. (2015) report that both GO and rGO exhibit dose-dependent toxicity in glioma cells. Consistently, our study also revealed the toxic effects of GO and rGO *in vitro* and *in vivo*. Notably, we found that rGO exhibited higher toxicity than GO, indicating that the hydrophilic properties, smoother edges, and high oxygen content of GO reduce its cytotoxicity. However, the high affinity to the cell membranes of rGO may induce cell apoptosis by interfering with cell metabolism (Jarosz et al., 2016).

Furthermore, this study investigated the mechanism of toxicity of GO and rGO in cardiomyocytes and tissues. It is well-known that overproduction of ROS can influence the stability of DNA and RNA and decrease the activities of SOD, CAT, and GSH-Px and increase MDA content, thereby resulting in cell oxidative damage (Schieber and Chandel, 2014). In addition, mitochondrial dysfunction is closely associated with the generation of ROS and the change of mitochondrial membrane potential, which can induce cell apoptosis (Kim et al., 2004). Previous studies have proved that the toxic effects of GFNs are closely related to lipid peroxidation and oxidative stress induced by the generation of ROS. For example, Zhang et al. (2012) have reported that GO treatment significantly increases ROS level, decreases SOD activity, and induces MDA production in HeLa cells. Also, Chatterjee et al. (2014) have shown that both GO and rGO can induce oxidative stress by increasing the production of ROS in HepG2, which may be the major cause of cytotoxicity. Additionally, mitochondrial activity is also considered as the direct influence factor of GO that induces cell apoptosis or necrosis (Lammel et al., 2013). It has been proved that graphene can destroy the mitochondrial membrane potential and increase intracellular ROS, thereby triggering cell apoptosis through the mitochondrial pathway in human breast cancer cells (Gurunathan et al., 2013). In this study, we also found that both GO and rGO increased the contents of MDA and ROS, decreased the activities of SOD, CAT, and GSH-Px and lowered the mitochondrial membrane potential in H9C2 cells, which indicated that both GO and rGO induced oxidative damage and destroyed mitochondrial activity *in vitro*. Consistently, our *in vivo* results revealed that GO or rGO treatment led to lipid peroxidation and oxidative stress in myocardial tissues. These findings indicated that the cardiotoxicity of GO and rGO might be mediated by lipid peroxidation, oxidative stress, and mitochondrial dysfunction.

## Conclusion

In conclusion, we found that rGO could be generated by the reduction of GO following gamma irradiation, which showed higher cardiotoxicity than GO. Both GO and rGO exhibited cardiotoxicity by mediating lipid peroxidation, oxidative stress, and mitochondrial dysfunction. Previous reports revealed that β-estradiol or allopurinol treatment can be used for protection of cell and tissue damages against oxidative stress in H9c2 cells and cardiac tissues through upregulating heme oxygenase expression (Barta et al., 2018; Chen et al., 2019; Luo et al., 2020). It will be interesting to test whether related pharmacological interventions could alleviate the cytotoxicity of graphene family nanomaterials in the future.

## Data Availability Statement

The original contributions presented in the study are included in the article/supplementary material, further inquiries can be directed to the corresponding author/s.

## Ethics Statement

The animal study was reviewed and approved by The First Hospital of Jilin University.

## Author Contributions

JZ, H-YC, and J-QW performed the material preparation, data collection, and analysis. G-DW and LW wrote the first draft of the manuscript. All authors commented on previous versions of the manuscript, contributed to the study conception and design, and read and approved the final manuscript.

## Conflict of Interest

The authors declare that the research was conducted in the absence of any commercial or financial relationships that could be construed as a potential conflict of interest.
